# How Are Medicinal Plants Useful When Added to Foods?

**DOI:** 10.3390/medicines7090058

**Published:** 2020-09-14

**Authors:** Gema Nieto

**Affiliations:** Department of Food Technology, Food Science and Nutrition, Faculty of Veterinary Sciences, Regional Campus of International Excellence “Campus Mare Nostrum”, Espinardo, 30071 Murcia, Spain; gnieto@um.es

Consumers are concerned about the use of synthetic additives in foods and this has forced food processors to find ways to produce food products without the use of these additives. Therefore there is a need in the food industry to find “clean label” products. In this context, the inclusion of medicinal plants into food products is an excellent strategy to produce functional foods, because plant-based extracts are rich in phytochemicals, with particular importance due to the health-beneficial effects.

Therefore, it is reasonable to imagine that, over the next few decades, the uses of natural extracts from medicinal plants will rapidly increase. In this sense, the search for new, effective and cheap sources of bioactive compounds is attracting food industry interest. However, it is important to study the implication on sensory characteristics of natural extracts of medicinal plants on foods to select specific raw materials; some of them of residual origin are especially promising due to their lower costs. Natural extracts from medicinal plants can be used as effective strategies for the production of functional foods, therefore achieving a significant increase in social and environmental sustainability. However, extensive research on sensory characteristics, antioxidant and antimicrobial sources, optimization of concentration of the extracts and a better knowledge of the mechanisms of the implication on shelf life of food, is still required.

This Special Issue of Medicines presents the current state of the art in medicinal plants and foods, collecting original manuscripts focused on topic-related research. The main objective is to publish original research work related to the chemistry of medicinal plants, and their application in food systems. Research work related mainly to the antimicrobial, antioxidant, and anti-inflammatory activities of medicinal plants and their applications in food systems is also included. The papers contribute significantly to furthering scientific knowledge in the above-mentioned scientific fields.

This Special Issue gives an overview of the current knowledge and recent trends in the use of medicinal plants as antimicrobials and antioxidants in foods, their potentials and challenges. It is critical to understand the effect of natural extracts and optimize their combinations for use in food preservation in order to better exploit their synergistic effects against both lipid oxidation and spoilage of pathogenic organisms. These considerations warrant the introduction of new species with high phenolic compound contents for beneficial applications by the food industry.

Medicinal plants or medicinal herbs have been identified and used since ancient times to improve the sensory characteristics of food. The main compounds found in plants correspond to four major biochemical classes: Polyphenols, terpenes, glycosides and alkaloids. Plants synthesize these compounds for a variety of purposes, including protection of the plant against fungi and bacteria, defense against insects and attraction of pollinators and dispersal agents to favor the dispersion of seeds and pollens.

Nowadays, there is also a growing interest in medicinal plants as natural alternatives to synthetic additives in foods because herbs and spices are generally recognized as safe (GRAS) and are excellent substitutes for chemical additives. The major activities of extracts and herbs from medicinal plants are antimicrobial, anti-inflammatory, bactericidal, antiviral, antifungal and preservative for foods. The use of natural preservatives to increase the shelf life of food systems is a promising technology since many vegetal substances show antioxidant and antimicrobial properties.

Taking into account all these considerations, recent changes in legislation controlling the use of animal feed additives and increased demand by consumers for healthier food products, if possible free of chemical additives, have stimulated interest in bioactive secondary metabolites from medicinal plants as alternative performance enhancers.

Since ancient times, medicinal plants have been added to improve the sensory characteristics of food. These plants have been gaining importance in recent years as potential sources of natural food preservatives due to the growing interest in the development of safe and effective natural food preservation. The use of vegetal substances with antimicrobial and antioxidant properties to increase the shelf life in foods is a promising technology.

The objective of including functional ingredients into foods is not only concerned with providing it with certain desirable properties, but also an attempt to change its image in these health-conscious days. In the market of functional food, rapid progress has been made in the development of this kind of food, based on the results of studies on food components providing positive health benefits over and above normal nutritional benefits.

The reduction or elimination of synthetic antioxidants in the elaboration of food could shorten the shelf-life, and this is the main concern for its marketing. As an alternative to synthetic antioxidants, natural extracts can be used, from plants such as grape, olive, sesame seed, tea, soybean, rosemary, thyme, etc., with antioxidant properties. Antioxidant compounds are usually added at a moderate dosage level since high levels of inclusion may mechanistically cause adverse effects through pro-oxidative action.

Between the strategies used to include natural additives, food can be modified by external addition in the elaboration of these products or by the addition into the animal diet of these extracts, where these ingredients are able to eliminate or reduce components that are considered harmful.

Work from our laboratory provides enough evidence for the use of natural extracts from medicinal plants in food preservation. Different studies found several interesting compounds in the plants and demonstrated the antioxidant and antimicrobial activity of natural extracts.

Nieto et al. [1]. reviewed the antioxidant and antimicrobial properties of rosemary. This review gives an overview on the on the use of natural extract from rosemary as a preservative in foods. Their use is limited due to their negative organoleptic properties, such as odour and taste. However, different new extraction methods have been developed in order to get colourless and odourless rosemary extracts. Several studies have reported that bioactive compounds, present in rosemary extracts and essential oils, delay lipid oxidation and microbiological spoilage, extending the shelf life of food. Taking into account all these aspects, rosemary extracts could be used in functional foods, pharmaceutical products, plant products and food preservation. The application of this natural extract can be complimented in different food systems such as meat, oils and dressing.

In the current publication, Serrano et al. [2] reviewed the potential anti-inflammatory health effects of traditional herbs. This review includes a summary of the dose–response anti-inflammatory activity of little-studied traditional botanical species, such as citrus fruit extracts rich in hesperidin, camu-camu (*Myrciaria dubia*), blackcurrant (*Ribes nigrum*), devil’s claw (*Harpagophytum procumbens*) and cat’s claw (*Uncaria tomentosa*). All these extracts reported greater or similiar effects than tea and cocoa. In addition, the known pathways of action and the potential synergistic effects of the constituent compounds of the extracts were also discussed.

Chiocchetti et al. [3] studied five agro-industrial food by-products, such as rice bran, pumpkin, peels from cucumber, cupuaçu seed peel and jackfruit, in order to establish alternative sources of nutrients. They determined the macronutrients, total and dialyzable Fe, the concentrations of iron-uptake inhibitors (phytic acid, tannins, fiber) and their correlation with iron bioavailability, several compounds that are interesting antifungal agents. These authors concluded that some by-products, in particular cucumber and pumpkin peels, could be valuable alternative sources of bioavailable Fe to reduce iron deficiency in at-risk populations and may contribute significantly to iron intake. This study showed the search for new by-products that can be used as alternative and inexpensive iron sources, and research into the development of new products based on cucumber and pumpkin peel.

Ghout et al. [4] describe their work to find biologically-active compounds from two extracts of the plant species *Euphorbia dendroides* L. They describe several compounds that are interesting antiproliferative and antioxidant antifungal agents, such as chlorogenic and gallic acids as major compounds. The two extracts exhibited antioxidant and anticancer activity. Based on the total phenolics and flavonoids contents, they concluded that the two extracts of *Euphorbia dendroides* L. display important reducing capacity, lipid peroxidation and antiradical activities.

Agregan et al. [5] provide a study of the the antioxidant potential of extracts obtained from three brown macroalgae, such as *Fucus vesiculosus, Ascophyllum nodosum* and *Bifurcaria bifurcata*, and two microalgae, such as *Spirulina platensis* and *Chlorella vulgaris* using a green and innovative extraction (ultrasound-assisted extraction using water/ethanol). Among the obtained macroalgae extracts, Bifurcaria bifurcata and the other macroalgaes were particularly suitable to be used as sources of phenolic antioxidants and to be included in products for human consumption.

Lefahal et al. [6]. describe their work to find biologically-active compounds from crude methanolic extract of aerial parts of *Capnophyllum peregrinum* (L.) Lange (Apiaceae) growing in Algeria. They describe several compounds that are interesting antifungal agents. The methanolic extract was found to have high flavonoid and phenolic contents as well as photoprotective and antioxidant activities. They could be used a sunscreen in pharmaceutical or cosmetic preparations and as a natural source of antioxidants.

Grigorakis et al. [7] describe their work to find biologically-active compounds, such as polyphenol, from *Stachys mucronata* and study of its antiradical activity. These authors reported as major constituents: apigenin analogues, derivatives of the flavone luteolin, chlorogenate conjugates and flavonol glycosides, being the most potent radical-scavenging compounds detected in the n-butanol fraction of the extracts, suggesting that they are the most active antioxidants in *Stachys mucronata*. This study showed valuable data for future studies that will aim at investigating the possible biological effects of *Stachys mucronata*, which remain unexamined to date.

Martínez et al. [8] describe in their work the search for biologically-active compounds from hydroxytyrosol (HXT) and their possible uses as functional ingredients in meat. They expose the health benefits provided by HXT consumption and the latest research about its use on meat. Due to its molecular structure, its regular consumption has several beneficial effects such as antioxidant, anti-inflammatory, anticancer, and as a protector of skin and eyes, etc. For these reasons, the use of HXT extract is a good strategy for use in meat products to replace synthetic additives. However, this extract has a strong odour and flavour, so it is necessary to previously treat this compound in order to not alter the organoleptic quality of the meat product when is added as an ingredient. For that reason, researchers have currently been focused on the encapsulation of this extract and the production of emulsion gels to prevent the sensorial alteration of meat products. In addition, the inclusion on meat endogenously, through the animal diet, or through its application in new packaging systems are the best strategies in order to introduce it into meat products.

Jongberg et al. [9] study in their work the effects of phenolic extracts from green tea (*Camellia sinensis*) and maté (*Ilex paraguariensis*) on the oxidative stability of modified atmosphere pork chops. They showed that phenolic plant extracts could be added as antioxidants in meat to prevent lipid oxidation, but depending on the concentration applied, may affect proteins either through covalent interactions or by serving as a prooxidant. They studied the oxidative stability in chops cut from injection-enhanced loins containing three different levels of green tea or maté extract. They concluded that Maté is a good source of antioxidants for the protection of both lipids and proteins in brine-injected pork, though the dose must be carefully selected.

Jongberg et al. [10] study in their work the protein oxidation and sensory quality of brine-injected pork loins added ascorbate or extracts of green tea or maté during chill-storage in a high-oxygen modified atmosphere. They showed that green tea and maté were found to equally protect against lipid oxidation-derived off-flavors, and maté showed less prooxidative activity towards proteins as compared to ascorbate, resulting in more tender meat. Based on present results, maté extract could be a potential substitution for ascorbate in the production of brine-injected pork, more so than green tea extract, as maté extract did not affect protein cross-linking, tenderness, or juiciness negatively throughout storage. Compared to green tea, maté extract generated no off-flavor, and, hence, based on the findings, is a valuable alternative as an antioxidant in brine-injected meat.They reported that maté is a valuable substitute for ascorbate in brine-injected pork chops.

Shafiee et al. [11] study the determination of blood glucose lowering and metabolic effects of *Mespilus germanica* L. hydroacetonic extract on streptozocin-induced diabetic BALB/c mice. The serum glucose lowering, normalization animal body weight, and antioxidative stress effects of *Mespilus germanica* L. leaf extract were investigated in normal and streptozotocin-induced BALB/c mice. The present study indicated that the *Mespilus germanica* leaf extract significantly decreased serum glucose and maintained normal body weight in BALB/c diabetic mice as compared with control groups. In addition, this extract decreased oxidative stress and lipid peroxidation. In conclusion, this species and other citable plants are very valuable and should be evaluated in experimental and clinical trials for their pharmacological efficacy and the discovery of new approved drugs for diabetes mellitus.

Egbung et al. [12] investigated the cardioprotective and hypolipidemic activity of *Vernonia calvoana* extract in paracetamol-treated rats. They suggested that the ethanol leaf extract of *Vernonia calvoana* reported cardioprotective and lipid-lowering effects and is a strategy to manage the toxicities induced by paracetamol.

Jaradat et al. [13] investigated new antilipase agents from ten traditional Palestinian edible and medicinal plants through inhibition of the absorption of dietary lipids. This effect was compared to Orlistat. Among all the extracts studied, Vitis vinifera and Rhus coriaria had the highest antilipase effects and could be considered a natural inhibitors of the pancreatic lipase enzyme and to be an excellent treatment of obesity players in obesity. These plants can be consumed in the diet or be prepared as natural supplements to treat or prevent obesity and control weight gain, and can be used for the treatment of hyperlipidemia.

The purpose of the current writing is to publish original research work related to the chemistry of medicinal plants, and their application in food systems. The research works published are related mainly to the antimicrobial, antioxidant, and anti-inflammatory activities of medicinal plants and their applications in food systems. The author of the current editorial encourages the modern use of extract from medicinal plants after performing trials of stability and sensory appreciation into foods.
